# The first study on antimicrobial resistance of *Staphylococcus aureus* isolated from raw goat milk associated with subclinical mastitis in Siliragung Subdistrict, East Java, Indonesia

**DOI:** 10.14202/vetworld.2023.786-791

**Published:** 2023-04-18

**Authors:** Ratih Novita Praja, Aditya Yudhana, Amung Logam Saputro, Jonathan Mark Hamonangan

**Affiliations:** 1 Veterinary Medicine Study Program, Department of Health and Life Sciences, School of Health and Life Sciences, Universitas Airlangga, Wijaya Kusuma Street 113, Banyuwangi, East Java, Indonesia; 2 Department of Veterinary Science, Division of Veterinary Microbiology, Faculty of Veterinary Medicine, Universitas Airlangga, Kampus C Mulyorejo Street, Surabaya, East Java, Indonesia; 3 Department of Veterinary Science, Division of Veterinary Parasitology, Faculty of Veterinary Medicine, Universitas Airlangga, Kampus C Mulyorejo Street, Surabaya, East Java, Indonesia; 4 Department of Veterinary Science, Division of Veterinary Clinic, Faculty of Veterinary Medicine, Universitas Airlangga, Kampus C Mulyorejo Street, Surabaya, East Java, Indonesia; 5 Veterinary Medicine Study Program, School of Health and Life Sciences, Universitas Airlangga, Wijaya Kusuma Street 113, Banyuwangi, East Java, Indonesia

**Keywords:** antimicrobial resistance, food security, infectious disease, public health, tropical disease

## Abstract

**Background and Aim:**

Raw goat milk is a highly nutritious dairy product and a suitable medium for the growth of antimicrobial-resistant *Staphylococcus aureus*, the leading cause of subclinical mastitis. This study aimed to investigate the resistance status of *S. aureus* which isolated from goat milk associated with subclinical mastitis cases in Siliragung Subdistrict, Banyuwangi District, East Java, Indonesia.

**Materials and Methods:**

The *S. aureus* isolates were recovered from 258 raw goat milk samples from seven different dairy goat farms. Preliminary screening of subclinical mastitis using the California mastitis test, then samples with score +3 and +4 were taken for further isolation and identification, followed by a biochemical test to determine the *S. aureus*. Moreover, the bacteria susceptibility test against several antimicrobials was done using the disk diffusion method.

**Results:**

Based on our findings, a total of 66 (25.58%) raw goat milk samples were tested positive for *S. aureus*, of which 36.36% were identified as multidrug-resistant. Moreover, *S. aureus* were also identified as resistant to penicillin (81.82%), ampicillin (65.15%), erythromycin (50.52%), and gentamicin (36.09%).

**Conclusion:**

The prevalence of *S. aureus* isolated from raw goat milk associated with subclinical mastitis in Siliragung Subdistrict, Banyuwangi District, Indonesia, was recorded at 25.58%. Moreover, 36.36% of *S. aureus* isolates were categorized as resistant to three or more classes of antibiotics. The biosafety and biosecurity procedures during the milking process should be strengthened in dairy goat farms to prevent the transmission of antimicrobial resistance among animals, humans, and environments.

## Introduction

Raw goat milk is categorized as a highly nutritious dairy product, making it a suitable medium for the growth of microorganisms, mainly bacteria [1]. Microbial contamination is one of the primary sources of food contamination worldwide, and in Asian countries such as Indonesia [2–4]. *Staphylococcus aureus* is a foodborne pathogen of great importance to animal and human health [5]. It is responsible for the contamination of dairy products, such as raw and fresh milk, during their handling and processing, making them unhealthy for human consumption and a threat to individuals who routinely consume dairy products [6]. Raw milk, as a dairy product, might play a potential role in transmitting antimicrobial-resistant bacteria to human populations that consume these products [7]. The occurrence and importance of antimicrobial resistance in *S. aureus* are triggered by the increasingly extensive use of antibiotics because of improper treatment doses, especially when *S. aureus* becomes the causative agent of subclinical and clinical mastitis in dairy goats [8]. To date, awareness of antimicrobial-resistant *Staphylococcus* spp. has become the primary concern in public health because it affects not only animals but also milk products routinely consumed by humans [9]. In the dairy cattle industry, subclinical mastitis is caused by *Staphylococcus* spp. affects dairy production worldwide and is associated with the extensive use of several antibiotics [10]. However, studies on antimicrobial resistance in *S. aureus* associated with subclinical mastitis infections in goats still need to be expanded because most studies have focused only on the prevalence rate of subclinical mastitis cases without investigating the status of antimicrobial resistance in several causative bacteria. In addition, the findings of several studies are limited because the majority focused only on the isolation and identification of *Staphylococcus* spp. and other bacteria as infectious agents that can potentially cause mastitis [11–13]. However, the prerequisites for the prevention of bacterial transmission at farm or industry levels depend on identifying not only *Staphylococcus* spp. but also all causative agents of mastitis, with the priority being antibiotic susceptibility patterns [14].

The occurrence of antimicrobial resistance is detrimental to farmers and veterinarians and has a direct impact on the global economy [15, 16]. Bacterial contamination of dairy products may occur directly from infected animals or through other routes, such as cross-contamination during transport, storage, and processing [17]. In East Java, Indonesia, most dairy farming management is still categorized as conventional. Current farming management in East Java results in low-quality milk owing to bacterial contamination, which leads to subclinical mastitis. *Staphylococcus aureus* is frequently identified as the main causative agent, whereas *Streptococcus* spp., *Pseudomonas aeruginosa*, *Enterobacteriaceae*, *Mycoplasma* spp., and other pathogens occur at the lower frequencies [18, 19]. The most common route of *S. aureus* transmission is through milking management when the milker’s hands have direct contact with the teats of dairy cattle or goats [20]. Other sources of contamination are the water and production equipment used in the milking procedures [21]. The antimicrobial-resistant *S. aureus* obtained from raw milk samples plays a significant role because these pathogens can infect humans and calves, leading to the spread of this antimicrobial-resistant pathogen to humans and animals [22]. Therefore, antibiotic susceptibility determination in raw milk contaminating bacteria is necessary to monitor potential transmission sources during milking procedures and prevent the transmission of antimicrobial resistance genes among consumers.

To our knowledge, there are no reports of antibiotic-resistant *S. aureus* isolated from raw goat milk in East Java. Thus, this study aimed to investigate the occurrence of *S. aureus* as a potential agent for subclinical mastitis in dairy goats from several farms in the Siliragung Subdistrict, Banyuwangi District, East Java, Indonesia. Moreover, this study also assessed scientific information regarding the *S. aureus* susceptibility patterns to several antibiotics, including those frequently used in human and veterinary treatment, especially in East Java, Indonesia.

## Materials and Methods

### Ethical approval

Fresh milk samples were used in this study. Hence, ethical approval was not required for this study.

### Study period and location

The study was conducted from May to August 2022. Milk samples were collected from seven goat farms in the Siliragung Subdistrict, Banyuwangi District, East Java, Indonesia (latitude: –8.493277, longitude: 114.084479). Milk samples were aseptically collected from both teats (separate milk) of apparently-healthy lactating goats. Briefly, the teats were wiped with swabs soaked in 70% ethanol, and a few streams of milk were discarded. The 10–15 mL of milk was collected into a sterile tube, labeled, and immediately brought to the Laboratory of Microbiology, School of Health and Life Sciences, Universitas Airlangga. The samples were kept at 4°C, immediately checked for subclinical mastitis, and then cultured within 24 h of collection.

### Study design

A total of 258 raw goat milk samples were collected for this study. Initial screening for subclinical mastitis was performed using the California mastitis test (CMT) (Kruuse, UK). CMT scores were graded as +1, +2, +3, and +4 according to the degree of reaction. Milk samples with CMT scores of +1 and +2 were categorized as negative, while samples +3 or +4 were considered positive. Briefly, CMT was performed using 3–4 mL of raw goat milk, to which an equal volume of CMT reagent was immediately mixed by swirling/circular motion. The reaction was graded based on the intensity of gel formation and color change. Samples with scores of +3 and +4 were used for further isolation and identification of *S. aureus* [22]. Samples were streaked onto Mannitol salt agar (Merck KGaA, Germany) and incubated at 37°C for 24–48 h. Colonies showing typical and non-typical Staphylococcus characteristics were examined microscopically using Gram staining. Confirmed Staphylococcus genus isolates were further identified using a biochemical panel, including the following tests: catalase, oxidase (Millipore 1.00181.0002, Canada), Voges–Proskauer, coagulase, urease, and fermentation of mannitol to identify the species. Based on culture, Gram staining, and biochemical tests, *S. aureus* was the only species isolated from all goat milk samples.

### Antimicrobial susceptibility test

The antimicrobial susceptibility of *S. aureus* was determined by the disk diffusion method on Mueller−Hinton agar (Oxoid CMO337, UK). The antibiotic disks used were from Oxoid, with the following types and concentrations: penicillin G (P, 10 IU), gentamicin (GEN, 10 μg), ampicillin (AMP, 10 μg), ciprofloxacin (CIP, 5 μg), erythromycin (E, 15 μg), tetracycline (TE, 30 μg), trimethoprim (W, 5 μg), chloramphenicol (C, 30 μg), cephalothin (KF, 30 μg), and novobiocin (NO, 30 μg). The results were interpreted according to Clinical and Laboratory Standards Institute Criteria [23]. *Staphylococcus aureus* ATCC 25923 was used as a control. Isolates showing susceptibility to all antibiotics were considered sensitive and those resistant to three or more antimicrobial classes were considered multidrug-resistant (MDR) isolates.

### Statistical analysis

The experimental data in the present study were designed as tables and figures, then analyzed using a descriptive method. The percentage of MDR was obtained using the formula MDR = A/B, where A is the number of bacteria which categorized as resistant against three or more antibiotics tested and B is the total number of bacteria which performed for the sensitivity test.

## Results

**Figure-1:**
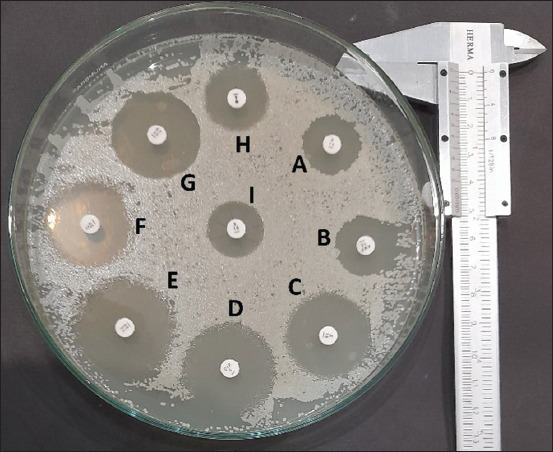
The result of the antibiotic diffusion test in a Petri dish with a diameter of 12 cm. (A) Penicillin indicated resistance, (B) ampicillin indicated resistance, (C) erythromycin indicated sensitive, (D) ciprofloxacin indicated sensitive; (E) tetracycline indicated sensitive; (F) chloramphenicol indicated sensitive; (G) cephalothin indicated sensitive; (H) trimethoprim indicated sensitive; (I) gentamicin indicated resistance.

A total of 66 (25.58%) raw goat milk samples tested positive for *S. aureus* (Table-1). All milk samples were collected from seven farms in the Siliragung Subdistrict, Banyuwangi. However, the total dairy goat population in each farm was different, that is 20 (farm 1), 46 (farm 2), 18 (farm 3), 50 (farm 4), 28 (farm 5), 32 (farm 6), and 64 (farm 7). Moreover, all farms still use conventional milking management and do not use a milking machine, so teats frequently have direct contact with the farmer’s hands. Farm 1 had the highest prevalence (40%), followed by farm 4 (32%), 5 (28.57%), 3 (27.77%), 2 (23.91%), and 7 (20.31%). The lowest prevalence was recorded in farm 6 (15.63%). Bacteriological tests of milk samples revealed yellow colonies with yellow zones in mannitol salt agar containing Gram-positive cocci in clusters that were, catalase-positive, oxidase-negative, Voges–Proskauer positive, coagulase-positive, urease-positive, and mannitol fermentation-positive. Based on this identification, the colonies were confirmed to be of *S. aureus* species. *Staphylococcus aureus* isolates were categorized as resistant to several antibiotics (Figure-1), and resistance to P was the highest (81.82%) among the other groups. Resistance to NO was the lowest (4.4%) among the other groups of tested antibiotics (Table-2). Moreover, 24 of the 66 *S. aureus* isolates were categorized as MDR. The highest percentage of MDR was recorded in farm 7 (38.46%), indicating that 5 of 13 *S. aureus* isolates were resistant to three or more antibiotic classes. The lowest MDR percentage was recorded in farm 3 (20%), with one out of five *S. aureus* isolates categorized as resistant to more than three classes of antibiotics (Table-3).

**Table-1:** Isolation and identification of *Staphylococcus aureus* from raw goat milk samples in Siliragung Subdistrict, Banyuwangi District, Indonesia.

Farm	Samples (n)	*Staphylococcus aureus* positive (n)	Percentage
1	20	8	40
2	46	11	23.91
3	18	5	27.77
4	50	16	32
5	28	8	28.57
6	32	5	15.63
7	64	13	20.31

**Table-2:** Essential information regarding *Staphylococcus aureus* resistance against the antibiotics tested.

Class of antibiotics	Groups of antibiotics according to their significant differences	Resistant to the antibiotic (%)	Intermediate to the antibiotic (%)	Sensitive to the antibiotic (%)
Beta-lactam	Penicillin	81.82	-	18.18
	Ampicillin	65.15	-	34.85
	Cephalothin	19.51	5.97	74.52
Aminoglycosides	Gentamicin	36.09	4.16	59.75
Quinolones	Ciprofloxacin	13.12	-	86.88
Macrolides	Erythromycin	50.52	2.51	46.97
Tetracyclines	Tetracycline	18.78	1.82	79.4
Sulfonamides	Trimethoprim	19.83	2.12	78.05
Chloramphenicol	Chloramphenicol	17	1.7	81.30
Other molecules	Novobiocin	4.4	-	95.60

**Table-3:** Multidrug resistance of *Staphylococcus aureus* from raw goat milk samples.

Farm	*Staphylococcus aureus* (n)	MDR *S. aureus* (n)	Percentage
1	8	3	37.5
2	11	3	27.27
3	5	1	20
4	16	6	37.5
5	8	4	50
6	5	2	40
7	13	5	38.46
Total	66	24	36.36

MDR=Multidrug resistance

## Discussion

The results of the study showed that the prevalence of *S. aureus* in Siliragung Subdistrict, Banyuwangi District, Indonesia, was 25.58%, which is higher than that reported in previous studies in Shaanxi, China (23.53%) [24] and the Oromia Region, Ethiopia (16.6%) [5]. However, it is slightly similar to a previous report from Northeast Brazil, which recorded a rate of 22.6% [14]. This high prevalence of subclinical mastitis may be due to improper farming management systems, such as poor milking management, poor environmental sanitation, and conventional milking procedures that lead to *S. aureus* transmission among dairy goats [18–21].

Based on our study, P was identified as the most resistant among the antibiotics tested and recorded at 81.82%, which is higher than that reported by Jamali *et al*. [20] and Silva Júnior *et al*. [14]. In contrast, NO was identified as the most sensitive among the antibiotics tested and recorded at 95.60%, followed by CIP (86.88%). Antibiotics such as KF (74.52%), W (78.05%), TE (79.4%), C (81.30%), CIP (86.88%), and NO (95.60%) were categorized as susceptible when tested against *S. aureus* isolates in this study. Penicillin (81.82%), AMP (65.15%), E (50.52%), and GEN (36.09%) were categorized as antibiotic-resistant.

Since 1942, *S. aureus* has been known to be resistant to P and more than 80% of individuals diagnosed with the infection of *S. aureus* strains were P-resistant by the late 1960s [25]. The present findings showed that 46.97% were still sensitive to E, 50.52% were categorized as resistant, and 2.51% as intermediate. In contrast, Harijani *et al*. [18] reported that 100% of the isolates were susceptible to E. Novobiocin has been proven to be effective in the treatment of *S. aureus* infections, in accordance with the studies of Mohamed *et al*. [26] and Tamendjari *et al*. [22].

The total percentage of MDR *S. aureus* isolates in our study was 36.36%. Treatment of bacterial infections is considered very difficult due to the rapid emergence of antimicrobial resistance, especially by multidrug strains, which are resistant to three or more classes of antibiotics, making antibiotic therapy difficult not only in animals but also in humans [24]. Most MDR isolates were suspected to be resistant to beta-lactams, macrolides, and aminoglycosides.

The most frequent manifestations of beta-lactam- mediated resistance in *S. aureus* are changes in membrane integrity and transmission of resistance genes from one strain to another. Inactivation of antibiotics by enzymatic (beta-lactamase) synthesis, target changes in penicillin-binding proteins, restriction of drug uptake by biofilm development (reduced drug uptake), and active efflux of the medication (drug efflux) are some of the biochemical mechanisms that cause beta-lactam resistance [27].

Macrolide resistance occurs by promoting the separation of peptidyl-tRNA molecules from ribosomes during elongation, thereby preventing the production of proteins. As a result, protein synthesis was irreversibly stopped, and the polypeptide chains were terminated. The adenine-N6 methyltransferase modified 23S rRNA post-transcriptionally, which was the first mechanism of macrolide resistance to be identified. These enzymes modify a single adenine in the 23S rRNA moiety by adding one or two methyl groups [28]. *Staphylococcus aureus* has two primary strategies for resisting macrolides: Alteration of the bacterial ribosome and macrolide efflux from the bacterial cell and ribosome protection through ATP-binding-cassette family proteins. Broad-spectrum resistance to macrolides is brought on by modification of the ribosomal target site, while efflux and enzymatic inactivation are less significant [29].

Aminoglycoside resistance mainly develops in *S. aureus* through enzymatic alterations. Enzymatic alteration of these antibiotics’ amino or hydroxyl groups is the primary mechanism underlying aminoglycoside resistance in *S. aureus* isolates [28]. In staphylococcal species, the enzyme inactivation of aminoglycoside-modifying enzymes is a vital resistance mechanism [30].

Antimicrobial resistance can spread through the consumption of food products of animal origin, making food a potential vector for antibiotic-resistant microorganisms. *Staphylococcus aureus* is thought to be a significant cause of zoonotic diseases and a potential source of transmission of antimicrobial-resistant strains between livestock and humans by handling and consuming contaminated food [22]. The outbreak of antibiotic-resistant bacteria, which is a challenge for the safety of food products, may be primarily caused by food items such as raw milk and dairy products made from raw milk. This issue is prevalent in the developing world because of poor food handling procedures, insufficient food safety rules, lax hygiene standards, a lack of funding for food safety investments, weak regulatory systems, and insufficient training for food handlers [31]. Poor management in farms and improper use of antimicrobial doses to treat the infection can be risk factors that lead to the transmission of antimicrobial-resistant bacteria. Antimicrobial resistance genes found in pathogenic bacteria can be horizontally transmitted from the environment to the raw milk. Antimicrobial drugs are widely used on dairy farms. Consequently, the raw milk microbiome can have a high resistance level. Because the bacteria in raw milk are not prevented from reproducing further without heat treatment, their resistance genes are amplified. The risk of horizontal gene transfer may increase due to increased antimicrobial resistance genes. These strains may come into contact with the human microbiota following the intake of animal products, and the conditions may be favorable for horizontal gene transfer-derived dissemination of antimicrobial resistance genes among these populations [32].

The prevalence of antibiotic-resistant bacteria in dairy goat farms can be reduced by routine and proper sanitation procedures. Antimicrobial-resistant bacteria may be present in raw milk if antibiotics are overused at therapeutic and sub-therapeutic levels in dairy goat farms. Therefore, the presence of antibiotic-resistant foodborne microorganisms in raw milk could put humans at risk for food safety if they are not heated [31]. Humans who consume raw milk can be infected with antimicrobial-resistant bacteria and antibiotic residues if the withdrawal time is not noticed.

Antibiotic residues have the potential to spread antimicrobial-resistant microorganisms, which can be directly transmitted to humans by ingestion. To reduce antibiotic residues in milk, a multifaceted strategy is needed, including education of milk producers, stricter regulation of antibiotic sales and withdrawal periods, heightened surveillance of residues and antimicrobial resistance in food animal products, and raised awareness and concern among policymakers and veterinary officials about antimicrobial resistance and its transmission pathways [33]. The challenge associated with considering mastitis from a One Health perspective revolves around the diversity of causative bacteria, especially antimicrobial-resistant species, which may impact the pathogenesis of mastitis. The normal bacterial content in milk is increasingly categorized as crucial for triggering immune development; thus, subclinical mastitis presents particular challenges in farms where inappropriate antibiotic therapy is likely to have profound effects on the microbiome [34]. In veterinary medicine, most studies on mastitis have focused on the pathogenic organisms involved, which have led to a better understanding of the epidemiology and pathogenesis of mastitis in the context of bacterial abundance. In addition, a new paradigm for mastitis was proposed based on the importance of host inflammatory mediators as potential disease drivers. Therefore, the One Health approach is relevant to the study of mastitis, which is related to the microenvironmental response to causative pathogens [35]. It is pertinent to emphasize that taking a pathogenic approach, such as with antimicrobial-resistant bacteria, will affect the whole environment and antibiotic therapy in cases of subclinical mastitis in dairy goat farms.

## Conclusion

This study concludes that *S. aureus* prevalence rate from raw goat milk associated with subclinical mastitis in Siliragung Subdistrict, Banyuwangi District, East Java, Indonesia, was recorded at 25.58%. Moreover, 36.36% of *S. aureus* isolates were identified as resistant to three or more classes of antibiotics. Results clearly indicated that raw goat milks were contaminated with the antibiotic resistance *S. aureus* which potentially infecting not only animals but also humans. One Health approach emphasized on preventive measures such as strengthening hygiene and sanitation during the milking process are required to reduce bacterial contamination. The high presence of MDR *S. aureus* raises questions about the persistent use of antibiotics as a main treatment for udder and other infections on dairy goat farm. Therefore, continuous monitoring and improvement of the hygienic quality of raw milk by ensuring proper handling and production to reduce the spread of MDR bacteria to foods of animal origin.

## Authors’ Contributions

RNP: Designed the study, materials preparation, data analysis, and manuscript preparation. AY and ALS: Conducted the surveys and participated in laboratory examinations. JMH: Contributed to the milk samples collection and field examination. All authors have read, reviewed, and approved the final manuscript.
